# Association between malnutrition exposure in early life and elevated atherogenic index of plasma in adulthood

**DOI:** 10.3389/fnut.2025.1542731

**Published:** 2025-05-20

**Authors:** Qiqi Lai, Rong Lian, Zhenghe Wang

**Affiliations:** ^1^Preventive Medicine, School of Public Health, Southern Medical University, Guangzhou, China; ^2^Department of Nephrology, Guangzhou First People’s Hospital, The Second Affiliated Hospital, School of Medicine, South China University of Technology, Guangzhou, China; ^3^Department of Epidemiology, School of Public Health, Southern Medical University, Guangzhou, China

**Keywords:** malnutrition exposure, early life, atherogenic index of plasma, Chinese population, the Great Chinese famine, age-balanced control group

## Abstract

**Background:**

Research on the relationship between malnutrition exposure in early life and atherogenic index of plasma (AIP) in adulthood is limited and whether this association exists in the Chinese population is unknown. This study was aimed to explore whether early exposure to the Great Chinese Famine is associated with elevated AIP in adulthood using a nationally representative sample.

**Methods:**

2,864 participants were chosen from the China Health and Retirement Longitudinal Study’s 2011 national baseline survey, and all of them were categorized into preschool-exposed, infant-exposed, fetal-exposed, and non-exposed groups by birthdate. AIP was calculated by lg(TG/HDL-C). Logistic regression models were used to investigate the association between malnutrition exposure in early life and elevated AIP in adulthood. The influence of age difference was further reduced by using an age-balanced control group.

**Results:**

In comparison to the non-exposed group, the risk of elevated AIP in adulthood was higher in the fetal-exposed group (OR = 1.386, 95% CI: 1.073–1.791, *p* = 0.013). After considering for confounding variables, the fetal-exposed group still had a higher risk of elevated AIP in adulthood (OR = 1.887, 95% CI: 1.206–2.952, *p* = 0.005). Stratified analysis showed that the risk of elevated AIP in adulthood was higher in female participants (OR = 2.121, 95%CI: 1.163–3.867, *p* = 0.014) and participants from rural areas (OR = 1.786, 95%CI: 1.113–2.868, *p* = 0.016) in the fetal-exposed group. Similar associations were also observed taking the age-balanced control group as a reference.

**Conclusion:**

Fetal exposure to malnutrition might be associated with higher risk of elevated AIP in adulthood, especially in the female population and people who lived in the rural areas, indicating that they might have higher risk of cardiovascular diseases. Special attention and targeted intervention are needed for those who have experienced malnutrition in the fetal period, and AIP is expected to be an indicator for monitoring metabolism-related diseases for them.

## 1 Introduction

As the Developmental Origins of Health and Disease (DOHaD) hypothesis continues to gain traction (1, 2), a growing body of research has underscored the critical importance of early interventions during pregnancy. These studies emphasize that early-life nutritional status exerts a profound and lasting influence on long-term health outcomes (3). According to the World Health Organization (WHO), an estimated 149 million children under five years old were stunted (too short for their age) and approximately 45 million children were wasted (too thin for their height) in 2022 (4, 5). Globally, malnutrition in early life is particularly more prevalent in developing countries, such as China. As the United Nations Children’s Fund (UNICEF) reported, there were about 15 million children in China exposed to different degrees of malnutrition (6). Early-life malnutrition exposure cannot be ignored in the development of a country.

Numerous studies have indicated that early-life malnutrition is closely linked to the onset of various adult diseases, including cardiovascular diseases (7), diabetes (8), obesity (9), and other metabolic diseases (10). Malnutrition exposure in the early years of life not only affects body weight and growth rate (11), but also increases the risk of metabolic syndromes in adulthood by affecting insulin sensitivity and lipid metabolism (12). Bikov et al. (13) suggested that individuals with early malnutrition tend to exhibit high cholesterol levels and abnormal lipid metabolism in adulthood. Possible mechanisms may include affecting the expression of inflammatory factors in the body, the composition of cell membranes, the lipid anabolic pathways, and the composition of the gut microbiota (14, 15), which can lead to disorders of lipid metabolism in individuals. Among these factors, the imbalance between high-density lipoprotein cholesterol (HDL-C) and triglyceride (TG) is widely regarded as a critical risk factor for atherosclerosis, affecting cardiovascular health. A population-based cohort studies (16) have shown that long-term low HDL-C and high TG levels were strongly linked to the development of atherosclerosis and cardiovascular diseases.

Atherogenic index of plasma (AIP) is considered to be a significant indicator of lipid metabolism and arterial health and widely used to evaluate the cardiovascular diseases risk (17–19). AIP is calculated from the log-transformed ratio of TG (mg/dl) and HDL-C (mg/dl) in plasma (20, 21). Although individual lipid markers are also capable of reflecting lipid levels in the body, the utilization of AIP to calculate a combination of TG and HDL-C provides a better prediction of cardiovascular disease incidence (13, 22). Huang et al. (23) demonstrated that AIP was found to be positively relevant to the degree of atherosclerosis, and its elevation was often indicative of enhanced lipid deposition and inflammation in the arterial wall. Therefore, AIP is able to recognize the risk of atherosclerosis and related cardiovascular diseases at an early stage, even before the patient shows obvious symptoms. In recent years, research on the application of AIP in different populations, such as people with diabetes, people with hypertension, and people of different ages and genders, has shown that it has broad applicability and credibility and can be used for more comprehensive risk assessment (19, 24). Higher values of AIP usually indicate higher cardiovascular risk (25). The majority of studies (26–28) believed that: AIP less than 0.11 is low risk, AIP of 0.11 to 0.21 is moderate risk, and AIP more than 0.21 is high risk. Monitoring changes in AIP can help assess the effectiveness of therapeutic interventions, thereby providing a basis for individualized medical treatment and enhancing the relevance of clinical care.

Due to ethical constraints, we cannot directly conduct population experiments. Instead, the Great Chinese Famine (1959–1961) is a suitable natural experiment for exploring the influence of early malnutrition exposure (29). Nutritional deficiencies during this period not only impacted the health condition of individuals who were directly affected by the famine, but also affect the health of their offspring through inter-generational effects (30), who are more likely to develop metabolic syndromes such as hypertension and hyperlipidemia in adulthood (31). Although prior research has investigated the association between early-life malnutrition and the development of cardiovascular diseases (32), the specific relationship between early-life malnutrition and elevated AIP in adulthood remains inadequately explored. AIP, a straightforward and widely accessible biomarker, holds significant value in the assessment of cardiovascular risk. Nevertheless, further validation is required to ascertain its applicability to the Chinese population, particularly among individuals with a history of early-life malnutrition, and to elucidate its interactions with conventional risk factors such as smoking and alcohol consumption. We hypothesize that individuals exposed to early-life malnutrition will exhibit elevated AIP levels in adulthood. Consequently, AIP is anticipated to play a pivotal role in the early prediction of metabolism-related diseases within populations with a history of early-life malnutrition.

Therefore, with a combination of a large-sample cross-sectional study and a retrospective cohort study based on national baseline data from the China Health and Retirement Longitudinal Study (CHARLS), the present study investigated the potential impact of early malnutrition exposure during the Great Chinese Famine on elevated AIP in adulthood. We hoped to put forward new perspectives and data support for learning about the long-period implications of early-life malnutrition on arterial health, and to provide a scientific basis for the development of targeted nutritional intervention strategies. Moreover, we also hoped to exploit the relevance of AIP as a comprehensive marker of dyslipidemia and its potential to reflect the long-term metabolic impact of early-life malnutrition.

## 2 Materials and methods

### 2.1 Data source and study population

Our study was grounded in data from CHARLS 2011 national baseline survey, which targets people aged 45 years or older in randomly selected households. CHARLS employed a stratified four-stage cluster sampling method, which are essentially representative of China’s middle-aged and elderly population. In the first stage, 150 counties and districts, excluding Tibet, were sampled after successively ranking them by region, urban and rural areas, and GDP per capita. In the second stage, 3 primary sampling units (PSUs) were drawn from each counties or districts by applying proportional probability of size (PPS) and 450 villages/neighborhoods were sampled in total. In the third stage, 24 households were drawn from each PSU and 80 households were randomly selected from the sample frame using CHARLS-GIS software for those without PSUs, and a total of 10,257 households were randomly selected. In the fourth stage, one participant aged 45 years and above was randomly selected from each sampled household, for a final baseline population of 17,708.

**Figure 1:**
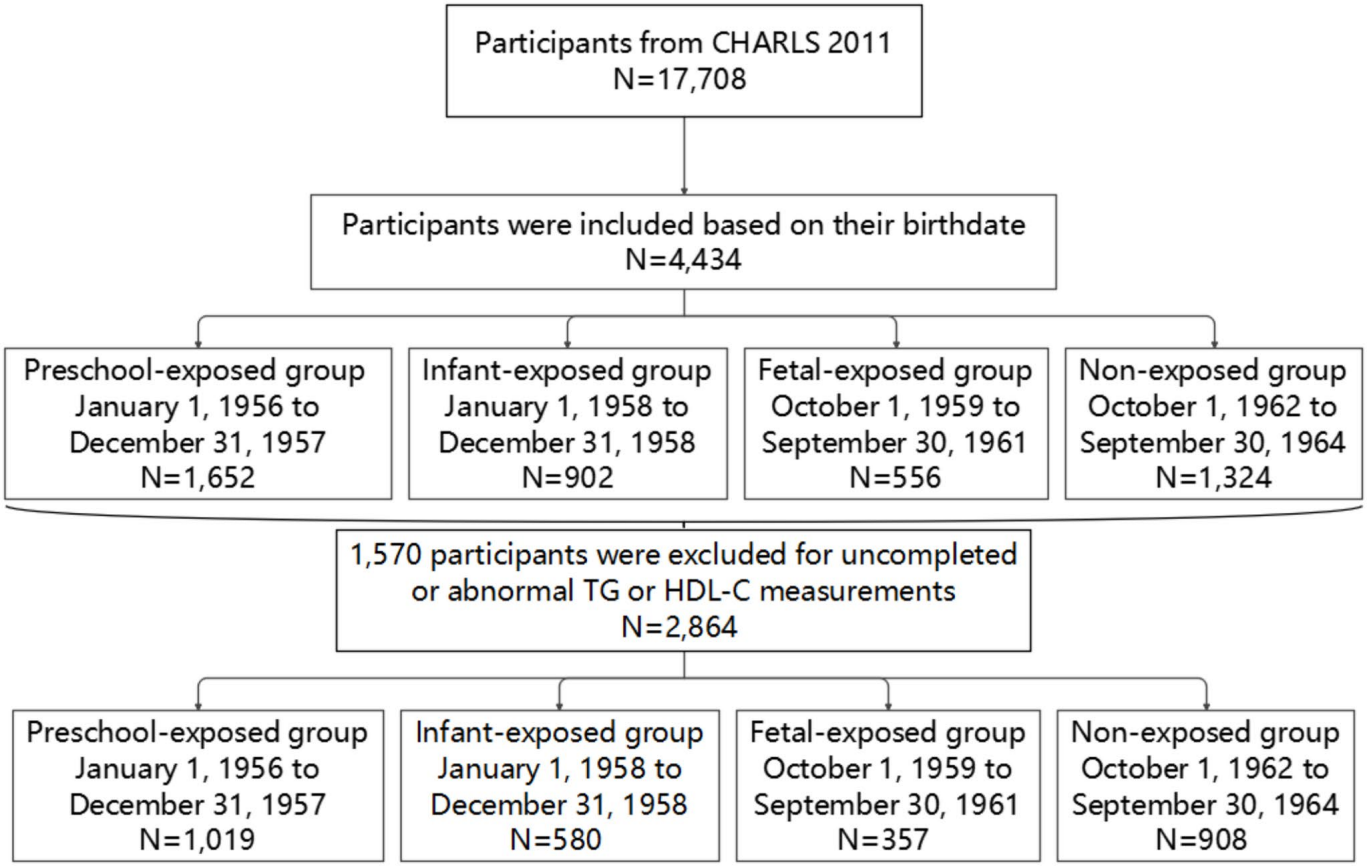
Flow diagram illustrating participant selection from the CHARLS 2011 cohort.

In terms of the selection of study participants, 4,434 participants were included based on their birthdate, after excluding 1,570 participants for uncompleted or abnormal TG or HDL-C measurements, 2,864 participants were enrolled into the study (Figure 1). All information was collected by uniformly trained interviewers, and each participant signed informed consent for all four parts of the questionnaire, physical examination, blood sample collection, and blood sample storage and analysis. CHARLS received approval from the Institutional Review Board of Peking University (IRB00001052-11015).

### 2.2 Classification of famine exposure groups

There are no official reports of the exact date from the beginning to the end of the Great Chinese Famine, so we consider the approximate time were 1959–1961. However, participants born between January 1, 1959 and September 30, 1959 were affected by famine both in fetal and infant stages, and participants born between October 1, 1961 and September 30, 1962 were exposed to famine both in fetal stage and non-exposed stage. To reduce classification error of exposure, these participants were eliminated from this study.

According to the date of birth, the participants were divided into four groups (33):

(1) Preschool-exposed group (January 1, 1956 to December 31, 1957),
(2) Infant-exposed group (January 1, 1958 to December 31, 1958),
(3) Fetal-exposed group (October 1, 1959 to September 30, 1961),
(4) Non-exposed group (October 1, 1962 to September 30, 1964).

### 2.3 Determination and calculation of AIP

AIP is a newly identified biomarker of atherosclerosis related to lipid metabolism, calculated as the logarithmic ratio of TG to HDL-C in the molar concentration. AIP is an indicator of the individual’s atherosclerosis risk. The level of AIP reflects the risk of cardiovascular diseases and atherosclerosis. The higher the value, the greater the risk. However, the risk classification of AIP has not yet been fully consistent in domestic and international research. The classification according to: AIP < 0.11 is low risk, AIP of 0.11–0.21 is moderate risk, and AIP > 0.21 is high risk of cardiovascular diseases, is widely recognized (34, 35). Consistent with most previously published epidemiological research (36, 37), participants were categorized into two distinct groups in this study based on the value of AIP: low AIP (< 0.11) and elevated AIP (≥ 0.11).

### 2.4 Covariates

In accordance with earlier studies, we integrated potential confounding factors at the baseline. Age (years), height (cm), weight (kg), waist circumference (WC, cm), body mass index (BMI, kg/m^2^), triglyceride (TG, mg/dl), and high-density lipoprotein cholesterol (HDL-C, mg/dl) were used as continuous variables. Height, weight and waist circumference were each measured twice. To calculate the BMI, the average values of these measurements were taken, using the formula BMI = weight (kg) / height (m)^2^. According to the value of BMI, participants can be divided into four groups: BMI < 18.5 for underweight, 18.5 ≤ BMI ≤ 23.9 for normal weight, 24 ≤ BMI < 27.9 for overweight, and BMI ≥ 28 for obesity (38).

Categorical variables such as gender, educational attainment, residence, smoking status, drinking status, AIP level, and physical activity (PA) level were utilized in this study. The gender was classified into male and female. The highest level of educational attainment was divided into four categories: primary school or below, middle school, high school, and college or above. Participants’ residence type was categorized as either rural or urban. Smoking status was divided into two groups: smokers and non-smokers. Drinking status was classified into three levels: drinking more than once a month, drinking less than once a month, and never drinking. The PA level was categorized into three groups: light PA, moderate PA, and vigorous PA (39).

### 2.5 Statistical analysis

Statistical analysis was conducted using SPSS 26.0 software. The Kolmogorov–Smirnov test was utilized to assess the normality of the measurement data. A *p*-value greater than 0.05 was considered indicative of a normal distribution. Continuous variables with a normal or approximately normal distribution were presented as mean ± standard deviation, and differences between multiple groups were compared using one-way ANOVA. For continuous variables that did not follow a normal distribution, the data was expressed as M (P25, P75) and the Kruskal-Wallis test was used for comparison of differences between multiple groups. Categorical variables were presented as relative numbers n (%) and differences between multiple groups were compared using the Chi-square test. A *p*-value less than 0.05 was considered statistically significant for all two-sided tests.

Logistic regression models were used to investigate the effect of malnutrition exposure at various periods of early life on elevated AIP in adulthood, compared with the non-exposed group. Factors to be adjusted for included residence, smoking status, drinking status, educational attainment, PA level, WC, and BMI. Stratified analysis was performed according to gender and residence to examine whether elevated AIP were affected by these factors.

Since the independent variable was grouped according to the birthdate of participants, which determined that the age of participants in four groups was statistically different. To minimize the impact of age differences between groups, we merged the non-exposed, infant-exposed, and preschool-exposed groups into a single age-balanced control group. This group was then used as a benchmark for comparing with the fetal-exposed group in order to examine the relationship between malnutrition exposure during this period and the likelihood of elevated AIP in adulthood.

## 3 Results

### 3.1 Baseline characteristic of the study population

Baseline characteristics, including age, gender, educational attainment, BMI, smoking status, residence type, WC, drinking status, PA level, and AIP level, were compared among four groups based on famine exposure. The results are presented in Table 1, which includes data from 2,864 middle-aged and older participants with a mean age of 52.24 ± 2.89 years. Of these participants, 1,292 (45.11%) were male and 1,572 (54.89%) were female. The non-exposed group had the youngest average age, while the preschool-exposed group had the oldest (*p* < 0.001). Significant differences were also found in the distribution of gender, educational attainment, BMI, and smoking status among four groups (*p* < 0.05). However, there was no statistical difference in the distribution of residence type, WC, drinking status, PA level, and AIP level (*p* > 0.05).

**Table 1:** Baseline characteristic of the study population.

Variables	Non-exposed group	Fetal-exposed group	Infant-exposed group	Preschool-exposed group	*p*-value
*N* (%)	1,019 (35.58)	580 (20.25)	357 (12.47)	908 (31.70)	
Age, years	48.99 ± 0.05	51.90 ± 0.06	54.05 ± 0.03	55.48 ± 0.04	<0.001
Height, cm	159.48 ± 0.29	159.60 ± 0.39	159.66 ± 0.47	159.22 ± 0.31	0.968
Weight, kg	62.09 ± 0.41	62.08 ± 0.56	61.02 ± 0.67	60.59 ± 0.41	0.074
WC, cm	84.00 ± 0.47	83.93 ± 0.65	83.95 ± 0.71	84.64 ± 0.44	0.930
BMI, kg/m^2^	24.33 ± 0.13	24.38 ± 0.21	23.90 ± 0.23	23.87 ± 0.29	0.021
TG, mg/dl	136.83 ± 3.42	150.79 ± 5.21	142.89 ± 7.46	136.13 ± 3.49	0.082
HDL-C, mg/dl	49.60 ± 0.44	49.29 ± 0.63	50.22 ± 0.80	50.38 ± 0.51	0.533
AIP	0.47 ± 0.02	0.48 ± 0.03	0.48 ± 0.04	0.45 ± 0.02	0.171
Gender, *n* (%)					0.003
Male	430 (42.20)	246 (42.41)	186 (52.10)	430 (47.36)	
Female	589 (57.80)	334 (57.59)	171 (47.90)	478 (52.64)	
Educational attainment, *n* (%)					<0.001
Primary or below	447 (43.87)	269 (46.38)	209 (58.54)	557 (61.34)	
Middle school	380 (37.29)	167 (28.79)	87 (24.37)	205 (22.58)	
High school	156 (15.31)	133 (22.93)	58 (16.25)	130 (14.32)	
College or above	36 (3.53)	11 (1.90)	3 (0.84)	14 (1.54)	
Residence type, *n* (%)					0.410
Rural	676 (66.34)	383 (66.03)	253 (70.87)	636 (70.04)	
Urban	120 (11.78)	78 (13.45)	38 (10.64)	119 (13.11)	
Smoking status, *n* (%)					0.003
Yes	339 (33.27)	202 (34.83)	147 (41.18)	365 (40.20)	
No	678 (66.54)	376 (64.83)	209 (58.54)	540 (59.47)	
Drinking status, *n* (%)					0.797
>once a month	275 (26.99)	159 (27.41)	102 (28.57)	242 (26.65)	
≤once a month	89 (8.73)	52 (8.97)	33 (9.24)	73 (8.04)	
Never	652 (63.98)	367 (63.28)	221 (61.90)	590 (64.98)	
PA level, *n* (%)					0.840
Light	111 (10.89)	76 (13.10)	39 (10.92)	117 (12.89)	
Moderate	134 (13.15)	80 (13.79)	43 (12.04)	120 (13.22)	
Vigor	181 (17.76)	111 (19.14)	52 (14.57)	168 (18.50)	
AIP level, *n* (%)					0.097
<0.11	239 (23.45)	105 (18.10)	78 (21.85)	200 (22.03)	
≥0.11	780 (76.55)	475 (81.90)	279 (78.15)	708 (77.97)	

WC, waist circumference; BMI, body mass index; TG, triglyceride; HDL-C, high-density lipoprotein cholesterol; AIP, atherogenic index of plasma; PA, physical activity.

### 3.2 Association between malnutrition exposure and AIP

Logistic regression models were used to assess the risk of elevated AIP in adulthood for the fetal-exposed, infant-exposed, and preschool-exposed groups, compared with the non-exposed group (Table 2). In model 1, without adjusting for any covariates, the fetal-exposed group had a higher risk of elevated AIP in adulthood (OR = 1.386, 95% CI: 1.073–1.791, *p* = 0.013). After adjusting for residence type and waist circumference in model 2, the risk remained higher in the fetal-exposed group (OR = 1.544, 95% CI: 1.151–2.072, *p* = 0.004). In model 3, after further adjusting for residence type, smoking status, drinking status, educational attainment, physical activity level, body mass index, and waist circumference, the risk of elevated AIP in adulthood was still higher in the fetal-exposed group (OR = 1.887, 95% CI: 1.206–2.952, *p* = 0.005). No significant effect was found in the infant-exposed and preschool-exposed groups (*p* > 0.05).

**Table 2:** OR (95%CI) for elevated AIP in adulthood among famine-exposed group.

Model	Non-exposed group	Fetal-exposed group	Infant-exposed group	Preschool-exposed group
Model 1	Reference	1.386 (1.073–1.791)*	1.096 (0.820–1.465)	1.085 (0.876–1.343)
Model 2	Reference	1.544 (1.151–2.072)**	1.165 (0.842–1.612)	1.140 (0.897–1.449)
Model 3	Reference	1.887 (1.206–2.952)**	1.058 (0.634–1.766)	1.068 (0.745–1.532)

Model 1 did not adjust for any covariate. Model 2 adjusted for residence type and WC. Model 3 further adjusted for smoking statues, drinking statues, educational attainment, PA level, and BMI. **p < 0.01, *p < 0.05.

### 3.3 Stratified analysis

In Table 3, the stratified analysis was conducted on factors such as gender and residence type to further investigate whether the risk of elevated AIP in adulthood was influenced by these factors. Stratification by gender revealed that only females in the fetal-exposed group had an increased risk of elevated AIP (OR = 2.121, 95%CI: 1.163–3.867, *p* = 0.014). Additionally, participants from rural areas in the fetal-exposed group were also found to have a higher risk of elevated AIP (OR = 1.786, 95%CI: 1.113–2.868, *p* = 0.016).

**Table 3:** Stratified analysis for elevated AIP in adulthood among famine-exposed group [OR (95%CI)].

Factors	Non-exposed group	Fetal-exposed group	Infant-exposed group	Preschool-exposed group	*p*-value for intervention
Gender					0.369
Male	Reference	1.778 (0.886–3.568)	0.850 (0.413–1.749)	1.326 (0.742–1.749)	
Female	Reference	2.121 (1.163–3.867)*	1.401 (0.633–3.103)	0.933 (0.581–1.498)	
Residence type					0.720
Rural	Reference	1.786 (1.113–2.868)*	0.980 (0.569–1.687)	1.082 (0.738–1.586)	
Urban	Reference	2.624 (0.584–11.787)	1.627 (0.276–9.599)	0.799 (0.261–2.443)	

All analyses were adjusted for residence type, smoking statues, drinking statues, educational attainment, PA level, BMI, and WC. *p < 0.05.

### 3.4 Analysis between age-balanced group and fetal-exposed group

In Table 4, the non-exposed, infant-exposed, and preschool-exposed groups were incorporated into one age-balanced control group, which was used as a reference to analyse the risk of elevated AIP in adulthood after 1 year of exposure to malnutrition in the fetal period. The results showed that the fetal-exposed group had a significantly higher risk of elevated AIP in adulthood compared to the age-balanced control group (OR = 1.822, 95% CI: 1.211–2.739, *p* = 0.004). Similar associations were observed in the female group (OR = 2.109, 95% CI: 1.208–3.683, *p* = 0.009) and the rural group (OR = 1.734, 95% CI: 1.127–2.669, *p* = 0.012).

**Table 4:** The risk of elevated AIP in adulthood compared with age-balanced group.

Category	Age-balanced group	Fetal-exposed group		
	Mean age (years)	Mean age (years)	OR (95%CI)	*p*-value
Total	52.34 ± 0.07	51.90 ± 0.06	1.822 (1.211–2.739)	0.004
Male	52.58 ± 0.10	51.94 ± 0.05	1.625 (0.875–3.019)	0.124
Female	52.14 ± 0.09	51.79 ± 0.04	2.109 (1.208–3.683)	0.009
Rural	52.37 ± 0.07	51.80 ± 0.04	1.734 (1.127–2.669)	0.012
Urban	52.20 ± 0.16	52.11 ± 0.08	2.648 (0.671–10.446)	0.164

All analyses were adjusted for residence type, smoking statues, drinking statues, educational attainment, PA level, BMI, and WC.

## 4 Discussion

The current study found that individuals who were exposed to malnutrition during the fetal period have a significantly higher risk of elevated AIP in adulthood compared to those who were not exposed to malnutrition, which was particularly pronounced in the female population and in the rural areas. These findings could potentially contribute to the development of public health policies by revealing the importance of early nutritional interventions and providing better health guidance strategies for populations growing up in a similar historical background. The correlation between early-life malnutrition and AIP suggests that AIP can be used as an early observable with the potential to reflect the long-term metabolic impact of early-life malnutrition. From a clinical perspective, the identification of AIP as a potential early marker of metabolic dysfunction offers a practical tool for risk stratification in populations with historical malnutrition exposure. The integration of AIP measurement into routine health screenings could enable timely identification of high-risk individuals, allowing for targeted lifestyle interventions or medical management before overt cardiovascular disease develops. This approach would be particularly valuable in resource-limited settings where advanced diagnostic capabilities may be scarce. In this way, the risk of cardiovascular diseases in adulthood would be able to gradually controlled through early prevention and intervention.

These results can be explained by the theory of the developmental origins of health and disease. According to this theory, nutritional and environmental exposures during pregnancy can increase the risk of chronic disease in adulthood for the fetus. As Hoffman et al. (40) suggested, the intrauterine growth period was extremely important for lifelong health because of the rapid growth and development of fetal tissues, organs, and systems. The robust link between early nutritional deprivation and later atherogenic risk underscores the critical window of vulnerability during fetal development, where nutritional insults may permanently alter metabolic trajectories. Nutrition interventions during critical developmental periods could potentially disrupt the intergenerational transmission of metabolic risk, offering a cost-effective strategy for reducing the growing global burden of cardiovascular diseases. Research has shown that social drivers, such as dietary norms and physical activity patterns, as well as broader ecological factors like pathogen burden and external mortality risk, can influence this life course trajectories (15). Therefore, further research is necessary to confirm the impact of acquired factors on this process. Additionally, any interference in this process due to nutritional deficiencies will not only interrupt or delay the growth process, but may also manifest as metabolic abnormalities in adult health, which may be linked to the role of cytokines (41, 42). Future research should focus on elucidating the precise biological mechanisms underlying this association and evaluating the effectiveness of targeted nutritional interventions at various life stages in mitigating these observed risks. The public health implications of these findings are profound, suggesting that investments in maternal and child nutrition programs may yield substantial long-term dividends in chronic disease prevention.

The gender disparity observed in our study, where females exposed to fetal malnutrition demonstrated a significantly higher susceptibility to elevated AIP in adulthood compared to males This finding was consistent with the results of a study by He et al. (3). The heightened risk among females may reflect the historical legacy of traditional Chinese gender norms, where patriarchal values systematically prioritized male offspring in resource allocation, creating a biological memory of nutritional injustice that manifests decades later in metabolic dysfunction. This phenomenon is supported by historical demographic data, which shows a sobering 7% excess female mortality during famines (43), serving as a stark numerical testament to the life-course consequences of gender-based nutritional discrimination. In addition to sociocultural explanations, the biological dimension presents equally compelling mechanisms. The accelerated developmental trajectory of females compared to males (44) suggests an earlier and potentially more vulnerable window for metabolic programming, where nutritional insults may have a more profound impact on developing physiological systems. Furthermore, the endocrine landscape, characterized by fundamental differences in hormonal regulation between sexes (45), may create divergent pathways for how early malnutrition translates to adult metabolic risk, with female physiology potentially exhibiting less buffering capacity against these early insults.

As for the residence type, the correlation between malnutrition exposure in fetal stage and higher risk of elevated AIP in adulthood was only observed in the rural areas. The occurrence of the Great Chinese Famine (1959–1961) was closely related to multiple factors such as policy failures, natural disasters, and unequal distribution of food between urban and rural areas, resulting in widespread famine. It was analogous to the results of Zhang et al. (30) that rural populations were disadvantaged in terms of social, economic and healthcare resources. In addition, rural areas suffered more from the famine as a result of the scarcity of food availability and limitations on urban–rural migration. Therefore, when providing health guidance and intervention strategies for populations growing up in similar context, the more affected rural areas should be highlighted (46).

There were some advantages in our study. First of all, the sample used was selected from the CHARLS database, which utilized a stratified four-stage cluster sampling method. This method ensured that the data was more representative of the middle-aged and elderly population in China. Additionally, the data collection process was carried out by professionals and trained personnel, including standard questionnaires, physical examinations, and laboratory tests. Furthermore, to account for potential confounding factors, the relationship between early-life malnutrition exposure and the risk of elevated AIP in adulthood was stratified by gender and residence type. To minimize the impact of age differences, the non-exposed, infant-exposed, and preschool-exposed groups were combined into one age-balanced control group, which allowed us to examine whether the association still existed in the fetal-exposed group. Moreover, a retrospective cohort design was utilized in our study, which allowed for a chronological sequence in examining the relationship between malnutrition exposure in early life and the increased risk of elevated AIP in adulthood. The study contributes meaningfully to the developmental origins of health and disease paradigm while providing actionable insights for clinical practice and health policy formulation, opening new avenues for preventive cardiology and personalized medicine approaches.

However, the current study also had some limitations. Firstly, this study used the Great Chinese Famine as a model for malnutrition exposure among participants, but famine was a natural event and population selection bias was inevitable. Secondly, the Great Chinese Famine lasted for 3 years, but without definite date boundaries. It was difficult to discriminate between the straightforward effects of the famine at different stages of early life. Thirdly, although participants in non-exposed group had not experienced famine themselves, their parents had experienced famine before pregnancy. Previous studies (29, 47) had shown that it was more possible for children of malnourished mothers to be affected and experience health problems, which might subsequently affect individuals throughout the life cycle. It was also possible that the level of AIP may be related to their parents’ experience of famine exposure, so further in-depth exploration of the specific mechanisms by how early-life malnutrition affects AIP in adulthood is needed.

In the current study, stratified analysis was conducted for the residence type of participants, which was divided into rural and urban. However, different regions and provinces in China were impacted to different degrees by the Great Chinese Famine. The severity of the famine was difficult to ascertain, and the degree of malnutrition exposure in early life was not the same. While we did control and adjust for various covariates, there may still be some unmeasured potential confounding factors such as postnatal nutritional levels, which might alter or even reverse the effect of malnutrition exposure during the fetal period (15). Therefore, in order to fully comprehend the health risks associated with early malnutrition, more comprehensive information and larger size of follow-up cohorts are needed.

## 5 Conclusion

In our investigation, we discovered that exposure to malnutrition during the fetal period is linked to a higher risk of elevated AIP in adulthood, especially in the female population and in the rural areas. As a result, it is crucial to prioritize the health concerns of individuals who have experienced malnutrition in their early years. Early nutritional interventions should be implemented, and AIP may serve as a practical biomarker that reflects the impact of malnutrition on metabolic dysfunction in the early stage.

## Data Availability

Publicly available datasets were analyzed in this study. This data can be found at: https://charls.pku.edu.cn/ (CHARLS).
